# Pharmacologic Targeting of miR29b with Bortezomib and Sorafenib to Improve Decitabine Sensitivity in Patients with Acute Myeloid Leukemia: Results from a Phase 1 Dose-Escalation Trial

**DOI:** 10.3390/cancers18010045

**Published:** 2025-12-23

**Authors:** Shivani Handa, Kristin Koenig, Qiuhong Zhao, Alice S. Mims, Sumithira Vasu, Ramiro Garzon, Tamanna Haque, Don Benson, Rebecca B. Klisovic, Guido Marcucci, Alison R. Walker, Bhavana Bhatnagar

**Affiliations:** 1 The Ohio State University Comprehensive Cancer Center, Columbus, OH 43210, USA; shivani.handa@osumc.edu (S.H.); qiuhong.zhao@osumc.edu (Q.Z.); alice.mims@osumc.edu (A.S.M.); sumithira.vasu@osumc.edu (S.V.); don.benson@osumc.edu (D.B.); 2 Cleveland Clinic, Taussig Cancer Institute, Cleveland, OH 44106, USA; kristin.koenig.13@gmail.com; 3 Huntsman Cancer Institute, University of Utah, Salt Lake City, UT 84112, USA; ramiro.garzon@hci.utah.edu; 4 Memorial Sloan Kettering Cancer Center, New York, NY 10065, USA; haquet1@mskcc.org; 5 University Hospitals Seidman Cancer Center, Cleveland, OH 44106, USA; rebecca.klisovic@uhhospitals.org; 6 The City of Hope Comprehensive Cancer Center, Duarte, CA 91010, USA; gmarcucci@coh.org; 7 Moffitt Cancer Center, Tampa, FL 33612, USA; alison.walker@moffitt.org; 8 West Virigina University Cancer Institute, Wheeling Hospital, Wheeling, WV 26003, USA

**Keywords:** acute myeloid leukemia, micro-RNA, miR29b, decitabine, bortezomib, sorafenib, epigenetic therapy

## Abstract

Background: Decitabine efficacy in acute myeloid leukemia (AML) may be enhanced by the pharmacologic upregulation of microRNA miR-29b, a regulator of DNA methyltransferase (DNMT) expression. Bortezomib and sorafenib have been shown preclinically to increase miR-29b levels, providing a biologically informed strategy to sensitize leukemic blasts to DNMT inhibition. Objectives: To evaluate the safety, tolerability, biological activity, and preliminary efficacy of combining bortezomib and sorafenib followed by decitabine in patients with newly diagnosed or relapsed/refractory AML. Methods: This phase I, dose-escalation study enrolled 15 patients (11 untreated, 4 relapsed/refractory) who received fixed-dose bortezomib and sorafenib across three dose levels prior to decitabine. Dose escalation was guided by dose-limiting toxicities (DLTs) and an increase in miR-29b expression. Results: The regimen was generally well tolerated with the most frequent grade ≥3 adverse events of hypertension and febrile neutropenia. At the highest dose level, a ≥2-fold increase in miR-29b expression was observed in two of the six evaluable patients. The overall response rate was 33.3%, with clinical responses observed in both newly diagnosed and relapsed/refractory patients. However, changes in miR-29b expression did not consistently correlate with clinical response. Conclusions: Sequential treatment with bortezomib and sorafenib followed by decitabine is feasible and demonstrates acceptable safety in AML. Although the biologic modulation of miR-29b was variable, this trial provides a proof of concept for pharmacodynamic-guided dose finding in epigenetic therapy combinations.

## Simple Summary

Decitabine is a commonly used drug for acute myeloid leukemia (AML), an aggressive blood cancer, but it does not work equally well for all patients. Our study tested whether giving two other approved drugs, bortezomib and sorafenib, before decitabine could make the leukemia cells more sensitive to treatment. The goal was to raise the levels of a genetic regulator called microRNA-29b, which has been linked to better responses to decitabine. In this early-phase clinical trial, 15 patients with newly diagnosed or relapsed AML were treated with increasing doses of this drug combination to identify the dose that best increased microRNA-29b levels. The treatment was generally safe, and about one-third of patients showed improvement in their leukemia. However, increases in microRNA-29b were not consistently linked to treatment response. A key strength of this study is its use of a biological marker to guide drug dosing, an approach that may help improve future AML treatments.

## 1. Introduction

Acute myeloid leukemia (AML) is a heterogeneous disease of the hematopoietic system characterized by a hierarchical expansion of immature myeloid cells in the blood and bone marrow, with each clone defined by unique genetic, epigenetic, and mutational features [1]. The presence or absence of specific chromosomal abnormalities or molecular mutations at diagnosis provides valuable prognostic information and also guides the selection of therapy. The recent approvals of small molecule inhibitors targeting mutations in *IDH1/2*, *FLT3*, and *KMT2A* rearrangements underscore the significance of understanding and therapeutically targeting mechanisms of leukemogenesis in order to improve outcomes in patients with this disease [2,3,4]. However, given that not all patients will have a targetable mutation, additional therapies are needed. One potential strategy is to augment the activity of an agent known to be clinically effective in AML.

DNA-hypomethylating agents such as azacitidine and decitabine are commonly utilized in the treatment of older patients with AML, given their tolerability and efficacy [5]. Our group previously reported a complete remission (CR) rate of 47% and an overall response rate (ORR) of 64% in newly diagnosed AML patients greater than 60 years of age who were treated with 10 days of decitabine [6]. Interestingly, higher pretreatment levels of the microRNA miR-29b were associated with clinical response to decitabine, suggesting its potential use as a predictive biomarker. MiR-29b is known to be involved in the regulation of DNA methyltransferase (DNMT) activity and is downregulated in leukemic blasts [7]. In vitro, the restoration of miR-29b expression reduces DNA hypermethylation by directly downregulating DNMT3a and 3b and indirectly targeting DNMT1 through the transcription factor Sp1 [8]. Based on these findings, we posited that the higher pretreatment miR-29b levels led to lower DNMT levels and increased sensitivity to the hypomethylating effects of decitabine.

Bortezomib is a dipeptidyl boronic acid proteasome inhibitor known to block proteasome–ubiquitin mediated intracellular protein degradation [9]. In vitro, bortezomib treatment increased miR-29b expression in AML cell lines via depletion of the transcription factor Sp1 and a decrease in Sp1/NF-κB complex binding [10]. However, a phase 2 study of decitabine in combination with bortezomib in AML patients older than age 60 did not find any significant improvement in overall survival compared to decitabine alone, suggesting that the additional effect of bortezomib with decitabine is insufficient to achieve this response [11]. Sorafenib is a multi-targeted kinase inhibitor known to be a potent inhibitor of VEGFR-2, PDGFRβ, KIT, FLT3, and p38 [12]. miR-155 is a microRNA known to be upregulated in AML with FLT3-ITD mutations, and its deregulation may result in epigenetic alterations via decreases in miR-29b expression [13]. We have found that sorafenib is effective in inhibiting miR-155 and increasing miR-29b expression in vitro. Sorafenib has been used in combination with decitabine to treat FLT3-mutated AML, though other FLT-3 targeted drugs are now available and preferred [14]. The combination of bortezomib and sorafenib with decitabine has not been previously investigated.

We hypothesized that preemptively restoring miR-29b in lower expressors via a pharmacological intervention causing miR-29b upregulation will lead to improved responses to decitabine. In this phase I trial, we explored the feasibility of a biologically driven strategy intended to increase miR-29b expression with sorafenib and bortezomib in order to potentiate decitabine activity in AML.

## 2. Methods

The study protocol was approved by the Institutional Review Board at The Ohio State University (OSU), USA on 9 April 2013 and is in accordance with the Declaration of Helsinki, the International Conference on Harmonization–Good Clinical Practice, and local laws. Informed consent was obtained from all patients. Patient recruitment, data collection, and all study-related procedures were conducted at the Ohio State University- James Cancer Hospital. All patients were followed for at least 30 days past the last dose of study drug. Patients removed from study for unacceptable adverse events were followed until the resolution or stabilization of the adverse event.

### 2.1. Patient Eligibility

Patients aged ≥60 years with previously untreated AML who were deemed to not be candidates for or who refused standard induction chemotherapy were enrolled. As the study progressed, enrollment was expanded to patients aged ≥18 years with relapsed or refractory AML. Patients with therapy-related AML or AML following an antecedent hematologic disorder (AHD) were eligible. Patients may have received growth factors, lenalidomide, 5-azacitidine, or the 5-day schedule of decitabine for an AHD. Patients who received the 10-day schedule of decitabine for treatment of an AHD were not eligible. Informed written consent approved by The Ohio State University Human Studies Committee was obtained prior to study entry. This trial was registered with the NCI clinical trials network (NCT01861314) on 20 May 2013.

Patients were required to have a total bilirubin <2.0 mg/dL, creatinine <2.0 mg/dL or CrCl ≥ 60 mL/min, ALT/AST <2.5 × the upper limit of normal (ULN), PT/INR <1.5 × ULN, and the Eastern Cooperative Oncology Group (ECOG) performance status 2. Patients with a pre-existing grade ≥ 2 neuropathy or other serious neurologic toxicity that would significantly increase the risk of complications from bortezomib therapy were excluded. Patients with acute promyelocytic leukemia were also excluded.

### 2.2. Study Objectives

The primary objective of the trial was to identify the biologically effective and tolerable dose (BETD) of the bortezomib/sorafenib combination in AML with biological activity defined as the dose(s) that induces a 100% increase (i.e., a doubling) in the level of miR-29b in bone marrow (bm) or peripheral blood (PB) after bortezomib/sorafenib treatment compared to pretreatment levels in at least five out of six patients at a given dose level. Defining the dose-limiting toxicities for sorafenib in combination with bortezomib/decitabine and identifying a recommended phase 2 dose level were the co-primary endpoints. The secondary objectives included overall response rate (ORR) and complete remission (CR) rates for this combination.

### 2.3. Study Design

**Figure 1:**
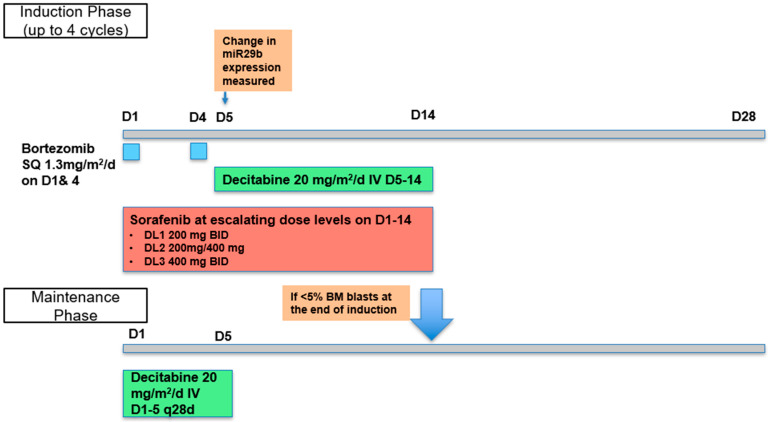
Treatment schema of induction and maintenance phase. Patients received bortezomib (D1 and 4) and decitabine (D5–14) at same dose level and sorafenib (given D1–14) was evaluated at three different dose levels.

The dosing of bortezomib and decitabine for each cycle of induction was the same for all dose levels (DLs), at 1.3 mg/m^2^/d subcutaneously (SQ) on days 1 and 4 and 20 mg/m^2^/d intravenously (IV) on days 5–14, respectively (Figure 1). The dose escalation of sorafenib on days 1–14 occurred at a dose of 200 mg bid at DL1, 400 mg/200 mg at DL2,, and 400 mg bid at DL3. Treatment was given in 28-day cycles and patients were eligible to receive up to four cycles of induction therapy. Bone marrow and peripheral blood samples were collected pretreatment and on day 5 of Cycle 1 to determine the change in miR-29b and pre-miR-29b expression. Patients who achieved less than 5% blasts in the marrow at the end of an induction cycle were eligible to receive maintenance treatment with decitabine, 20 mg/m^2^/d IV days 1–5 every 28 days. Patients could continue maintenance therapy until the development of unacceptable toxicity or disease progression.

To determine an optimal biologic and tolerable dose, the dose escalation was driven by both the incidence of dose-limiting toxicities (DLTs) and at least a 100% increase in mature or primary miR-29b expression from baseline in bone marrow or peripheral blood on day 5 of Cycle 1 (Supplementary Figure S1).

### 2.4. Response Criteria

The assessment of clinical response was made according to the International Working Group criteria [15]. Bone marrow aspirates and biopsies were performed prior to the initiation of Cycle 2 of induction in all patients; however, additional biopsies to assess treatment effect prior to Cycle 3 or 4 were only completed in the absence of circulating blasts in the peripheral blood. Of note, the blast percentage determination that allowed patients to proceed to maintenance chemotherapy was based on morphology only and did not consider flow cytometric or cytogenetic analysis.

### 2.5. Safety Assessments

Adverse events were graded according to the National Cancer Institute Common Terminology Criteria for Adverse Events (CTCAE) version 4.0. DLT was defined during Cycle 1. Grade 4 nonhematologic toxicities, with the exception of alopecia, nausea, and vomiting controllable with anti-emetic therapy, infection, and fatigue, were considered DLT. Toxicities that required temporary holding of sorafenib (but not permanent discontinuation) were not considered to be DLT unless the toxicity did not resolve to grade 2 or less by the end of the cycle. Patients who received at least 9 days of sorafenib (plus all doses of bortezomib and decitabine) were evaluable and not replaced unless the PI determined that additional patients at that dose level were required for safety purposes. Given the frequency of infectious complications associated with chemotherapy in AML, these were not considered DLT unless the severity or duration was longer than expected. Hematologic toxicity was defined as the failure to recover neutrophil and/or platelet counts by day 42 in patients with <5% blasts in the bone marrow, the absence of myelodysplastic changes, and/or the absence of evidence of disease by flow cytometry in the bone marrow.

### 2.6. Pharmacodynamic Analysis

The identification of the BETD level, defined by a 2-fold increase in *miR-29b* expression, was determined by RT-qPCR from baseline and from day 5 blood or marrow samples. We measured both the primary and mature transcripts of miR-29b; the former may offer a more direct and stable indicator of gene transcription, as mature miR-29b rapidly engages with the RNA-induced silencing complex. Baseline molecular studies, using next-generation sequencing with a targeted 96-gene panel to assess for the most common mutations in AML, were also performed on the Illumina Seq platform.

### 2.7. Statistical Methods

A biological dose-finding trial design was used in combination with the standard dose-limiting toxicity evaluation. Dose escalation was based on DLTs observed during the first cycle, and the rules of escalation are described in detail in the Supplementary Figure S1. The planned sample size ranged from 6 to 30 patients, consistent with a standard rule-based phase I design using cohorts of three to evaluate dose-limiting toxicities and determine the maximum tolerated dose. Dose selection also incorporated an optimal biologic dose-finding strategy as described by Hunsberger et al. [16]. A dose level was designated as the recommended phase II dose if fewer than two of six patients experienced dose-limiting toxicities and at least five of the six demonstrated biologic activity. Patient characteristics were summarized using the medians and ranges for continuous variables and frequencies and proportions for categorical variables. The response rate was calculated as the proportion of patients who achieved CR, CRi, or MLFS with 95% confidence intervals. The patients’ characteristics were compared between the responders and non-responders using Fisher’s exact test. The change in mir-29b was calculated as the difference at day 5 of treatment from baseline (ΔΔCT), and the fold change was calculated as 2^−ΔΔCT^. The overall survival (OS) was calculated from the start of treatment to the date of death, censoring those alive at the time of last contact. A Kaplan–Meier curve was generated, and the median OS was estimated. All the analyses were conducted using Stata 18 (College Station, TX, USA).

## 3. Results

### 3.1. Patients

**Figure 2:**
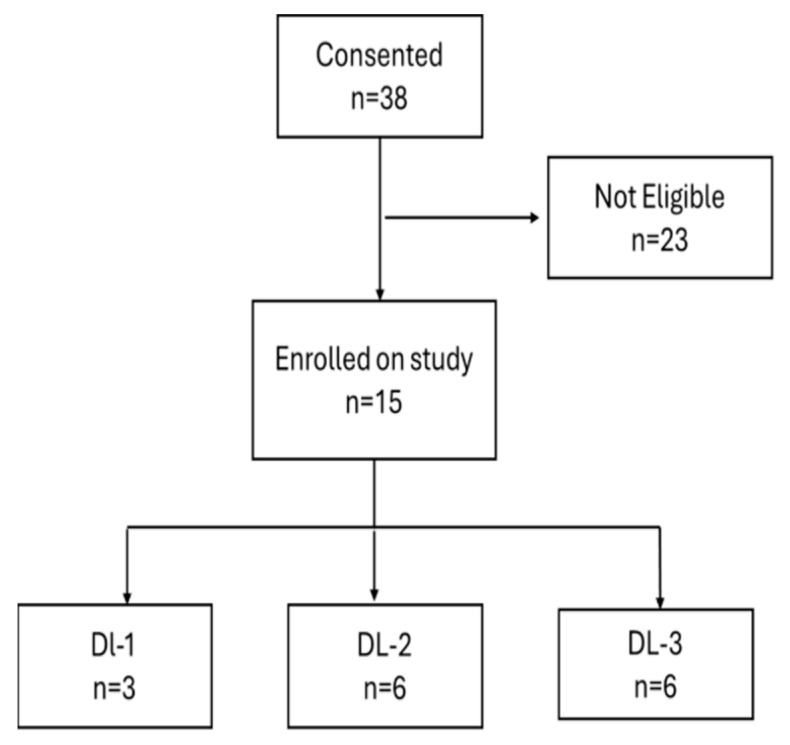
Consort diagram: In total, 38 patients consented to the study, of which 23 were found ineligible and 15 patients (11 newly diagnosed and 4 relapsed/ refractory) were enrolled at three escalating dose levels (DL 1–3).

The patient characteristics at all dose levels are shown in Table 1. Eleven patients with previously untreated AML and four patients with relapsed or primary refractory AML were enrolled between July 2013 to August 2015 (Figure 2). Both patients with relapsed AML had progressed in less than 6 months following reduced intensity allogeneic stem cell transplantation (SCT), and both patients with primary refractory AML were FLT3-ITD positive. Of the 11 patients with previously untreated AML, most (*n* = 9) patients were classified as intermediate risk, whereas one was classified as favorable and adverse risk, respectively, as per the 2017 European LeukemiaNet (ELN) classification [17].

### 3.2. Treatment and Toxicity

All nonhematologic toxicities, at least those possibly attributed to any of the investigational agents at any time during the treatment at all dose levels, are summarized in Supplementary Table S1. Febrile neutropenia occurred in ten patients, and infectious complications were common and included diverticulitis (n = 1), pneumonia (n = 5), and catheter-related infections (n = 6) (Table 2). These episodes were not more severe or prolonged than expected and did not meet the criteria for DLT. Additional grade 3 nonhematologic toxicities that were common included hypertension (n = 11) and QTc prolongation (n = 3), both requiring dose modifications of sorafenib. Grade 3 sensory or motor neuropathy attributable to bortezomib was not observed. Grade 1 and 2 peripheral neuropathy was reported in one patient each, respectively.

Three patients enrolled in DL A1. Two patients were removed after completing less than three cycles of induction chemotherapy, one because of progressive disease, and one withdrew in order to proceed with hospice care. There were no DLTs and no increase in miR-29b expression and therefore dose escalation to DL A2 occurred. Of the three patients enrolled, one patient developed grade 2 palmar–plantar erythrodysesthesia that prevented the administration of a sufficient number of doses of sorafenib. Though this did not meet the pre-defined DLT criteria, a decision was made to further explore the occurrence of this toxicity, and three additional patients were enrolled. None experienced this toxicity and only one received fewer than four cycles of treatment and was removed because of progressive disease. There were no DLTs at DL A2 and no increase in miR-29b expression. Three patients were enrolled to DL A3, of which one patient was removed after the completion of Cycle 1 because of disease progression. There were no DLTs, and correlative analysis revealed that two of the three patients exhibited more than a 100% increase in pri-miR-29b. Given this finding, three additional patients were enrolled with no additional DLTs.

### 3.3. Clinical Responses

**Figure 3:**
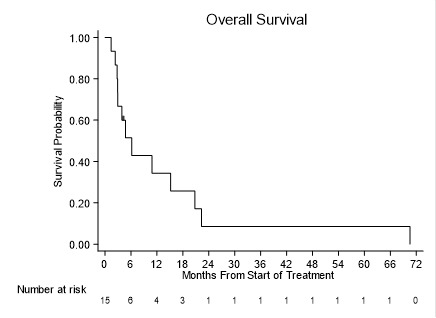
Overall survival (OS) of patients treated with bortezomib, sorafenib, and decitabine combination, including newly diagnosed and relapse/refractory AML patients. Median OS was 6 months (95% C.I.: 2.7–20.7 months).

Of the 15 patients enrolled, three patients achieved a CRi, and one patient each achieved a CR and MLFS, respectively, for an overall response rate (ORR) of 33.3% (Table 3). One patient who achieved a CRi did achieve platelet count recovery but refused to undergo a repeat bone marrow biopsy. Responses occurred in two patients with de novo AML, one patient with untreated secondary AML following MDS, and two patients with relapsed AML post-allogeneic stem cell transplant (alloSCT). All responders had a normal karyotype, and 4/5 responders had a *FLT3* mutation (3 *FLT3*-ITD and 1 TKD, respectively). The patient with the most durable response had normal karyotype *NPM1/IDH2* mutated, *FLT3*-negative AML, and achieved a CRi after one cycle of induction and went on to receive fourteen cycles of maintenance chemotherapy. Two additional patients received maintenance treatments of shorter durations with one and two cycles only, respectively, received. Interestingly, one of the patients who had relapsed after alloSCT achieved a CRi after three cycles of induction and went on to receive a donor lymphocyte infusion (DLI) and remained in remission for 5 years. Neither patient with primary refractory AML responded to therapy. The median overall survival (OS) for the entire cohort was short, at 6 months (95% C.I.:2.7–20.7 months), with only one patient (described above) alive after 5 years (Figure 3).

### 3.4. Correlative Studies

#### 3.4.1. Change in miR-29b Expression

**Figure 4:**
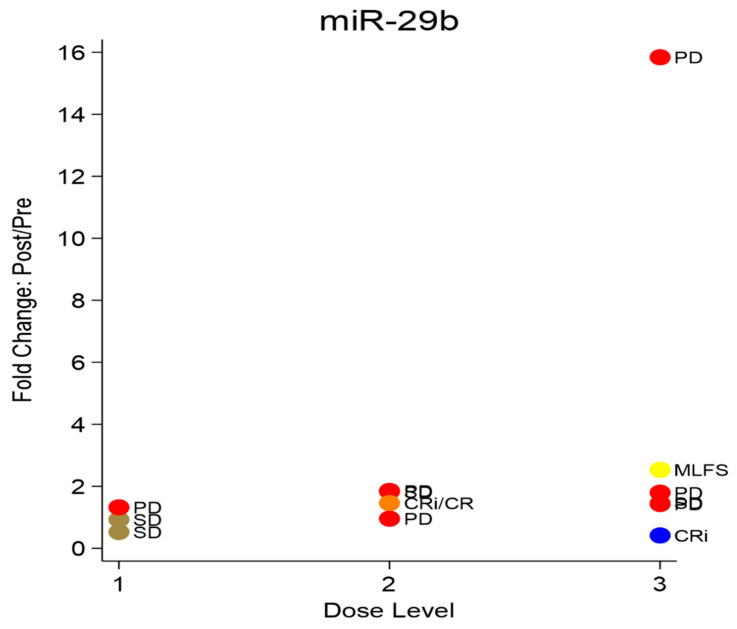
Correlation of change in pri-miR29b expression with clinical response per dose level. No clear correlation was seen in miR29b expression with increasing sorafenib dose levels. PD = progressive disease, SD = stable disease, CR = complete remission, CRi = complete remission with incomplete count recovery, and MLFS = morphologic leukemia free state.

Fourteen patients were evaluable for miR-29b expression level at day 5 relative to baseline (Supplementary Table S2, Figure 4). Nine of these fourteen patients (69%) had an increase in miR-29b expression, but only two achieved a two-fold or higher increase in pre-miR-29b expression. Data was available for four of the five treatment responders (CR, CRi, and MLFS). Two patients (with CR and CRi) had a reduction in miR-29b expression level, one patient with CRi had an increase but not a doubling, and one patient with MLFS had a 2.5 times increase in miR-29b expression level at day 5 compared to baseline. Ironically, the one patient with the highest level of increase in miR-29b expression (~15 times) did not achieve a response and had progressive disease.

Dose levelBest responseRQ1PD1.3211SD0.5251SD0.9262CR0.8332PD1.8532PD0.9562CRi/CR1.4592SD1.8183CRi0.4143SD1.4663PD1.7943MLFS2.533PD1.433PD15.84

#### 3.4.2. Molecular Studies

All patients had baseline *NPM1*, *FLT3,* and *CEBPA* testing results available, and 11/15 patients had a complete myeloid panel next-generation sequencing testing performed on baseline marrow samples. Patients were found to have between one and fifteen distinct subclonal mutations in their AML. Data was available for four of the five treatment responders (CR, CRi, and MLFS). Two of these responders were noted to have *IDH2* mutations, and the other two responders were noted to have *NPM1* and *FLT3* mutations. Three of the five patients with PD were noted to have a *RUNX1* mutation. No association was observed between mutation type and response (Table 4).

## 4. Discussion

The therapeutic strategies for patients with newly diagnosed, as well as relapsed/refractory AML, continue to evolve from a standard approach for all patients to one in which individual disease and patient characteristics guide the treatment choice. In addition to recurrent molecular mutations, intracellular pathways that promote proliferation, survival, and mechanisms of resistance within leukemic blasts are also potential pharmacologic targets. The development of strategies that are able to manipulate these pathways may improve the effectiveness of standard treatment options for patients.

This unique phase I trial evaluated the feasibility of enhancing endogenous miR-29b expression using a combination of sorafenib and bortezomib, with the goal of augmenting clinical responses to decitabine in patients with AML. We employed a biologically driven dose-escalation design, incorporating both dose-limiting toxicities (DLTs) and pharmacodynamic changes in miR-29b to identify the optimal dose. Notably, two patients at dose level 3, in which sorafenib was administered at the highest dose of 400 mg twice a day, exhibited a 100% increase in primary and mature miR-29b expression. Of the two patients with a biologic response, only one—who demonstrated a 2.5-fold increase in pri-miR-29b—achieved a clinical response (MLFS) whereas, unexpectedly, the patient with the highest (15-fold) increase had a progressive disease. This questions our central hypothesis and highlights a “biomarker” paradox. While the exact reasons for this disconnect between target engagement and clinical response are unclear, one potential reason maybe the use of bulk mononuclear cells (MNC) rather than sorted CD34+ cells for miR-29b assessment, which introduces a potential confounding effect related to early blast clearance. In responding patients, as leukemic blasts are eliminated, the measured miR-29b signal in bulk samples may decrease even in the setting of effective target engagement, and apparent decline in miR-29b should not be interpreted as a failure of pharmacologic modulation. Conversely, large increases in the miR-29b observed in non-responders may reflect the persistence of leukemic blasts rather than a biologically meaningful drug effect. Future studies should incorporate cell-subset-specific analyses, such as sorted CD34^+^ leukemic populations or single-cell approaches, to more accurately evaluate target engagement in dynamic disease settings like AML. Another limitation of our study is that we did not directly assess the downstream effects of miR-29b modulation on its known targets, including DNMT3a, DNMT3b, and DNMT1 (via Sp1), which are thought to mediate sensitivity to decitabine [8]. As a result, potentially unanticipated effects of the sorafenib and bortezomib combination on global DNA methylation patterns may have occurred but were not captured in this analysis. And finally, the biggest limitation of our study is the small numbers which make the results skewed by outliers.

The observed CR/CRi rate of 27% is lower than in our prior phase II trial, likely due to the small sample size and limited decitabine exposure (one-third of patients received only 1–2 cycles) [6]. However, other studies have reported similar response rates (~30%) with 10-day decitabine regimens, which aligns with our findings [18,19]. When given as single agents, neither bortezomib nor sorafenib have demonstrated significant clinical activity in AML [20,21]. While the addition of bortezomib to decitabine has not improved outcomes in prior studies, sorafenib combined with decitabine has shown some promise in small series, though it has not been evaluated in randomized trials [11,14]. We also observed a high rate (20%) of grade 4 sepsis similar to the higher infection rates reported in studies comparing 5 d vs. 10 d decitabine schedules [22]. Cardiovascular toxicity with a 73% rate of grade 3 hypertension and QTc prolongation from sorafenib was also notable. While our approach of repurposing approved agents to upregulate miR-29b was biologically rational and demonstrated a dose–response effect, with increased expression observed only at the highest dose level of sorafenib, the clinical landscape has shifted, with more potent, safer, and selective FLT3 inhibitors such as gilteritinib and quizartinib now favored over sorafenib. Additionally, Bcl-2 inhibitor venetoclax has become the standard of care in combination with hypomethylating agents for newly diagnosed AML in unfit patients and 10 d decitabine monotherapy is seldom utilized [22,23,24]. Future studies of Bcl-2 inhibitors and newer-generation FLT3 inhibitors should consider integrating miRNA expression profiling to identify potential biomarkers of response.

Our study underscores the challenges of translating microRNA biology into therapeutic advances. Despite the known prognostic or predictive roles of several microRNAs in AML (e.g., miR-126, miR-155, miR-181a/b, miR-25, miR-362, etc.), none have been successfully leveraged as direct therapeutic targets yet [25,26]. Other strategies to simultaneously modulate the kinome and epigenome—such as HDAC inhibitors that enhance miR-29b via disruption of the HDAC/Sp1-NF-κB complex—have also failed to demonstrate clinical benefit, as seen in prior trials combining vorinostat with bortezomib and sorafenib [27]. The direct modulation of microRNA expression through synthetic mimics or antisense oligonucleotides may be a more effective therapeutic strategy, though efficient delivery to the bone marrow remains a major obstacle [28,29]. A transferrin-conjugated nanoparticle delivery system for synthetic miR-29b has shown preclinical promise in murine AML models, and clinical evaluation is anticipated [30]. Most recently, a first in-human trial of 5-FU-miR-15a, a synthetic miR-15a mimic conjugated with cytotoxic pyrimidine analog 5-fluorouracil (5-FU), demonstrated safety and disease stabilization in patients with relapsed/refractory AML in preliminary results [31].

## 5. Conclusions

This phase I study demonstrates that sequential treatment with bortezomib and sorafenib followed by decitabine is feasible and tolerable in patients with newly diagnosed or relapsed/refractory AML. Using a biologically driven dose-escalation design, we identified a regimen that achieved pharmacologic modulation of miR-29b in a subset of patients, supporting the concept that endogenous microRNA expression can be altered through combination therapy. The overall response rate was modest and changes in miR-29b expression did not consistently correlate with clinical outcomes, which is a major translational barrier to this approach.

Importantly, the primary significance of this trial lies in its innovative design, integrating real-time pharmacodynamic markers to guide dose selection, rather than relying solely on toxicity endpoints. While the evolving treatment landscape of AML and the availability of newer targeted agents limit the direct clinical application of this regimen, the study provides proof-of-concept for biologically informed trial designs in epigenetic therapy. Future studies incorporating more potent targeted agents with a better safety profile or direct microRNA-based therapeutics may further refine this strategy and improve outcomes for patients with AML.

## Figures and Tables

**Table 1:** Baseline patient characteristics (*N* = 15).

	All (*n*= 15)	Dose Level 1 (*n* = 3)	Dose Level 2 (*n* = 6)	Dose Level 3 (*n* = 6)
**Age (years)**				
**Median**	71	75	74	60.5
**Range**	42–83	70–80	67–83	42–78
**Sex**				
**Male**	6	2	1	3
**Female**	9	1	5	3
**Race**				
**White**	15	3	6	6
**Ethnicity**				
**Non-Hispanic**	15	3	6	6
**Disease Type**				
**Relapsed**	2	0	0	2
**Refractory**	2	0	0	2
**Untreated**	11	3	6	2
**ELN category**				
**Favorable**	1	0	1	0
**Intermediate**	12	3	4	5
**Adverse**	2	0	1	1
**History of CNS disease**				
**No**	15	3	6	6
**History of extramedullary disease**				
**No**	15	3	6	6
**FLT3-Mutation**				
**No**		3	5	0
**Yes**	8	0	1	6
**CEBPA-Mutation**				
**No**	14	3	5	6
Yes	1	0	1	0
NPM1 Mutation				
**No**	10	3	3	4
**Yes**	5	0	3	2
**Prior MDS**				
**No**	12	2	4	6
**Yes**	3	1	2	0
**Prior MPN**				
**No**	14	2	6	6
**Yes**	1	1	0	0
**Baseline BM cellularity**				
**Median**	80	90	80	75
**Range**	25–95	70–95	25–90	30–95
**Baseline BM blast%**				
**Median**	43	59	38.5	59
**Range**	17–86	24–86	17–72	17–84

Abbreviations: ELN = European LeukemiaNet, CNS = central nervous system, BM = bone marrow, MDS = myelodysplastic syndrome, and MPN = myeloproliferative neoplasm.

**Table 2:** Incidence of grade 3/4 hematological and infectious toxicities.

	Grade 3 (*n*, %)	Grade 4 (*n*, %)
Anemia	15 (100%)	0
Thrombocytopenia	1 (7%)	13 (87%)
Leukopenia	4 (27%)	10 (67%)
Lymphopenia	11 (73%)	1 (7%)
Neutropenia	1 (7%)	13 (87%)
Febrile neutropenia	10 (67%)	0
Sepsis	0	3 (20%)
Pneumonia	5 (33%)	**0**
Urinary Tract infection	4 (27%)	0
Catheter related bloodstream infection	6 (40%)	0
Infective myositis	1 (7%)	0

**Table 3:** Clinical responses by dose levels.

Response	All (n = 15)	Dose Level 1 (n = 3)	Dose Level 2 (n = 6)	Dose Level 3 (n = 6)
PD	7	1	2	3
SD	3	2	1	1
CR/CRi	4	0	3	1
MLFS/<5% Blasts	1	0	0	1

Abbreviations: PD = progressive disease, SD = stable disease, CR = complete remission, CRi = complete remission with incomplete count recovery, and MLFS = morphologic leukemia free state.

**Table 4:** Association between mutation status and response.

	Non-Responder (*n* = 10)		Responder (*n* = 5)		
	***n***	**%**	***n***	**%**	***p*-Value**
***FLT3* mutation present**					0.58
No	6	60	1	20	
Yes	4	40	4	80	
***NPM1* mutation present**					0.12
No	8	80	2	40	
Yes	2	20	3	60	
***CEBPA* mutation present**					0.99
No	9	90	5	100	
Yes	1	10	0	0	
***IDH1* and/or *IDH2* mutation present**	(*n* = 7)		(*n* = 4)		0.49
No	1	14	2	50	
Yes	6	86	2	50	

## Data Availability

The original contributions presented in this study are included in the article/Supplementary Materials. Further inquiries can be directed to the corresponding authors.

## Supplementary Materials

The following supporting information can be downloaded at: https://www.mdpi.com/article/10.3390/cancers18010045/s1. Figure S1: Schematic for determination of BETD level; Table S1: Incidence of non-hematological toxicities; and Table S2: Fold-change in pri-miR29b expression on Day 5 as compared to baseline per dose level.
