# Supplementary material for: Pharmacologic Targeting of miR29b with Bortezomib and Sorafenib to Improve Decitabine Sensitivity in Patients with Acute Myeloid Leukemia: Results from a Phase 1 Dose-Escalation Trial
Source: Cancers (Basel). 2025 Dec 23;18(1):45. doi: 10.3390/cancers18010045 (PMC12784657; doi:10.3390/cancers18010045)
Supplement: Supplementary file 1 [file cancers-18-00045-s001.zip › cancers-3995856-supplementary.pdf]

Supplementary File

Table S1: Incidence of non-hematological toxicities

**Treatment-Emergent Adverse Event (N, %)**	**Grade 1 (reported in >10% pts)**	**Grade 2**	**Grade 3**	**Grade 4**
Constipation	9 (60%)	0	0	0
Hypokalemia	8 (53%)	1 (7%)	3 (20%)	0
Increased bilirubin	7 (47%)	1 (7%)	0	0
Increased alanine aminotransferase (ALT)	7 (47%)	0	0	0
Rash	6 (40%)	1 (7%)	0	0
Hypocalcemia	6 (40%)	4 (27%)	1 (7%)	0
Hyperglycemia	6 (40%)	5 (33%)	1 (7%)	0
Edema limbs	6 (40%)	1 (7%)	0	0
Hypomagnesemia	5 (33%)	0	0	0
Hyponatremia	5 (33%)	0	5 (33%)	0
Nausea	5 (33%)	0	1 (7%)	0
Musculoskeletal pain	4 (27%)	2 (13%)	0	0
Hypoalbuminemia	4 (27%)	7 (47%)	0	0
Fatigue	4 (27%)	4 (27%)	0	0
Diarrhea	4 (27%)	2 (13%)	2 (13%)	0
Cough	4 (27%)	1 (7%)	0	0
Increased aspartate aminotransferase (AST)	4 (27%)	2 (13%)	0	0
Anxiety	4 (27%)	1 (7%)	0	0
Hyperkalemia	3 (20%)	0	1 (7%)	0
Increased creatinine	3 (20%)	2 (13%)	1 (7%)	0
Epistaxis	3 (20%)	0	0	0
Vomiting	3 (20%)	1 (7%)	0	0
Hypophosphatemia	3 (20%)	1 (7%)	3 (20%)	1 (7%)
Hypotension	3 (20%)	0	1 (7%)	0
Insomnia	3 (20%)	1 (7%)	0	0
Urinary frequency	2 (13%)	0	0	0
Rectal pain	2 (13%)	0	0	0
Tinnitus	2 (13%)	0	0	0
Oral pain/ mucositis	2 (13%)	2 (13%)	0	0
Injection site reaction	2 (13%)	0	0	0

Hematuria	2 (13%)	0	0	0
Gastroesophageal reflux	2 (13%)	1 (7%)	0	0
QTc prolongation	2 (13%)	3 (20%)	3 (20%)	0
Dry mouth	2 (13%)	0	0	0
Bruising	2 (13%)	2 (13%)	0	0
Abdominal pain	2 (13%)	1 (7%)	0	0
Atrial Fibrillation	0	2 (13%)	0	0
Confusion	1 (7%)	2 (13%)	0	0
Generalized muscle weakness	0	2 (13%)	0	0
Hypertension	0	2 (13%)	**11 (73%)**	0
Urinary retention	0	2 (13%)	0	0
Ventricular Fibrillation	0	0	0	1 (7%)
Ventricular Tachycardia	0	0	0	1 (7%)

Table S2: Fold-change in pri-miR29b expression on Day 5 as compared to baseline per dose level.

Pt. No.	Dose level	Best response	ΔΔCт	RQ
1	1	PD	-0.402	1.321
2	1	SD	0.931	0.525
3	1	SD	0.111	0.926
4	2	CR	0.264	0.833
6	2	PD	-0.89	1.853
7	2	PD	0.065	0.956
8	2	CRi/CR	-0.545	1.459
9	2	SD	-0.862	1.818
10	3	CRi	1.273	0.414
11	3	SD	-0.552	1.466
12	3	PD	-0.843	1.794
13	3	MLFS	-1.340	2.530
14	3	PD	-0.520	1.430
15	3	PD	-3.985	15.840

ΔΔCT: post – pre miRNA expression level
RQ (RelativeQuantity): is 2^(-ΔΔCT)
MiR29b fold-change data was unavailable for pt #5
PD = progressive disease, SD = stable disease,
CR = complete remission, CRi= complete remission with incomplete count recovery, MLFS = morphologic leukemia free state

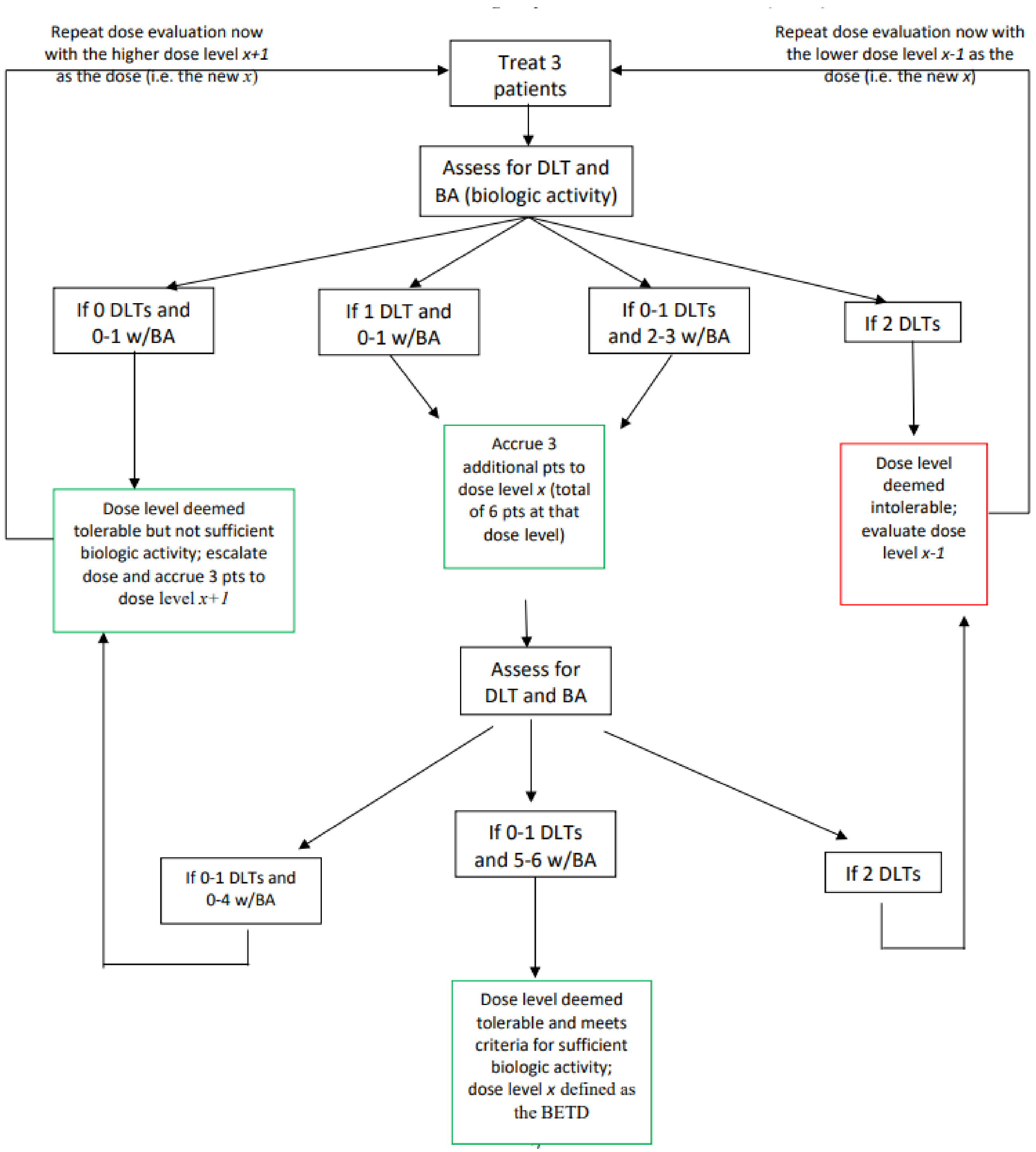

Supplementary Figure S1: Schematic for determination of BETD level
